# Transcriptional defects and reprogramming barriers in somatic cell nuclear reprogramming as revealed by single-embryo RNA sequencing

**DOI:** 10.1186/s12864-018-5091-1

**Published:** 2018-10-10

**Authors:** Yong Liu, Fengrui Wu, Ling Zhang, Xiaoqing Wu, Dengkun Li, Jing Xin, Juan Xie, Feng Kong, Wenying Wang, Qiaoqin Wu, Di Zhang, Rong Wang, Shaorong Gao, Wenyong Li

**Affiliations:** 1 Key Laboratory of Embryo Development and Reproductive Regulation of Anhui Province, Fuyang Normal University, Fuyang, 236041 Anhui Province China; 2 Clinical and Translation Research Center of Shanghai First Maternity & Infant Hospital, Shanghai Key Laboratory of Signaling and Disease Research, School of Life Sciences and Technology, Tongji University, Shanghai, 200092 China

**Keywords:** Transcriptional defect, Reprogramming barrier, SCNT embryo, RNA-seq, Mouse

## Abstract

**Background:**

Nuclear reprogramming reinstates totipotency or pluripotency in somatic cells by changing their gene transcription profile. This technology is widely used in medicine, animal husbandry and other industries. However, certain deficiencies severely restrict the applications of this technology.

**Results:**

Using single-embryo RNA-seq, our study provides complete transcriptome blueprints of embryos generated by cumulus cell (CC) donor nuclear transfer (NT), embryos generated by mouse embryonic fibroblast (MEF) donor NT and in vivo embryos at each stage (zygote, 2-cell, 4-cell, 8-cell, morula, and blastocyst). According to the results from further analyses, NT embryos exhibit RNA processing and translation initiation defects during the zygotic genome activation (ZGA) period, and protein kinase activity and protein phosphorylation are defective during blastocyst formation. Two thousand three constant genes are not able to be reprogrammed in CCs and MEFs. Among these constant genes, 136 genes are continuously mis-transcribed throughout all developmental stages. These 136 differential genes may be reprogramming barrier genes (RBGs) and more studies are needed to identify.

**Conclusions:**

These embryonic transcriptome blueprints provide new data for further mechanistic studies of somatic nuclear reprogramming. These findings may improve the efficiency of somatic cell nuclear transfer.

**Electronic supplementary material:**

The online version of this article (10.1186/s12864-018-5091-1) contains supplementary material, which is available to authorized users.

## Background

Utilizing somatic cell nuclear transfer (SCNT) technology, somatic cells have been reprogrammed not only to totipotency but also to form live cloned offspring [1]. Since the birth of “Dolly” [2], more than 20 species of mammals have been cloned from various somatic cells [3], including the cloned quadrumana [4], indicating the vast potential of SCNT technology in animal husbandry and endangered species conservation. Furthermore, nuclear transfer (NT) blastocysts have been used to produce NT embryonic stem cells [5], regardless of whether the NT blastocysts were obtained from a healthy control [6] or a patient [7]. Therefore, the SCNT technique also has wide prospective applications in human therapeutic cloning [8, 9].

Although the NT technique displays great potential in various applications, the developmental ability of NT embryos remains very poor [3]. The poor developmental ability is attributed to transcriptional defects and reprogramming barriers. A normal fertilized embryo undergoes a series of transcriptional regulatory steps after fertilization. The well-known transcriptional period ZGA is mainly regulated by maternal factors [10, 11] and epigenetic patterns [12, 13]. NT embryos appear to undergo abnormal gene regulation during this period. Certain somatic genes are constitutively expressed in 2-cell NT embryos [14]. Maternal factors are not degraded properly in NT embryos [15]. NT embryos display an abnormally high DNA methylation level in more than 20 genes [16]. Even the Oct4 regulatory region appears to exhibit an abnormally high methylation level [17, 18]. The level of methylation on H3K9 [13, 19] and H3K27 [20] is noticeably higher. This increased methylation also leads to the abnormal positioning of the PRC2 complex [20] and the abnormal expression of the polycomb-associated gene in NT embryos [21]. These epigenetic abnormalities are often regarded as reprogramming barriers. The abnormal epigenetic patterns further cause abnormal gene transcription in NT embryos [22, 23]. In conclusion, the abnormal transcriptional regulation in NT embryos may be responsible for their poor developmental ability.

Transcriptomic analyses are appropriate methods for studying transcriptional regulation. However, transcriptomic analysis of NT embryos is challenging to perform using the traditional transcriptomic analysis method, as a sufficient number of NT embryos are not typically accumulated for use in this analysis. Since the invention of single-embryo transcriptome analysis technology (RNA-seq) [24], many transcriptomic analyses of embryos have been successfully performed [25–28]. Several RNA-seq studies have focused on specific stages of NT embryos [13, 29], but no transcriptome blueprint describing the entire developmental period of NT embryos has been published. In the present study, the transcriptome blueprints of in vivo fertilized embryos (in vivo group), CCs donor NT embryos (NTC group) and MEF donor NT embryos (NTM group) were systematically analyzed using the RNA-seq method. Then, the differences in gene transcripts among these embryos and donor cells were extensively explored. This study provides a foundation for studies of the mechanism of nuclear reprogramming and promotes the application of NT technology in livestock production, therapeutic cloning and regenerative medicine.

## Methods

### Animals

All chemicals used in this study were purchased from Sigma (St. Louis, MO, USA), unless stated otherwise. The Institutional Animal Care and Use Committee of Fuyang Normal University approved this study. SPF grade C57BL/6 female mice and DBA2 male mice were purchased from the Model Animal Research Center of Nanjing University and housed at the Experimental Animal Center of Fuyang Normal University. B6D2F1 (C57BL/6 × DBA2) mice were bred at the Experimental Animal Center of Fuyang Normal University. All mice had free access to water and food and were maintained in an environment at 22–26 °C with 40–70% humidity on a 12/12 h light/dark cycle.

### Sources of oocytes and donor cells

The 8–10-week-old BDF1 female mice were superovulated with 7 IU of pregnant mare serum gonadotropin (PMSG), followed by 7 IU of human chorionic gonadotropin (HCG) by intraperitoneal injections after 48 h. The cumulus-oocyte complexes (COCs) were collected 14 h after HCG injection. COCs were digested in the M2 drop containing 0.1 mg/ml hyaluronidase. CCs were removed from the oocytes by gentle pipetting. The obtained denuded oocytes were used for NT after a 15 min incubation in G1 medium at 37 °C in a 5% CO_2_ atmosphere with saturated humidity. The remaining CCs were collected and temporarily stored at 4 °C until use as donor cells for NT. MEFs were obtained from 13.5-day-old BDF1 fetuses. MEFs were cultured in DMEM (Gibco, 11960–051) supplemented with 10% FBS. MEFs were used at passages 2–5 in this experiment. The cell cycle was synchronized to G0/G1 phase by culturing the cells in DMEM supplemented with 0.5% FBS for 72 h. Synchronized single-embryo MEFs were obtained by treating the cells with 0.25% Trypsin-EDTA (Gibco, 25300–072) and pipetting before use as donor cells for NT.

### Nuclear transfer

SCNT was performed using the micro-manipulator system. Oocytes were enucleated in an M2 drop containing 5 μg/ml cytochalasin B (CB). The spindle was removed using a microinjector that contained a glass pipette (inner diameter of 12 μm). The donor cells were transferred to M2 supplemented with 3% PVP, and the cell membranes were ruptured by several Piezo pulses. The donor nucleus was directly injected into the enucleated oocyte in M2 containing 5 μg/ml CB using a Piezo-driven pipette. The glass pipette used for the CCs had an 8-μm inner diameter, and the pipette used for the MEF cells had a 10-μm inner diameter. Before activation, the reconstructed embryos were incubated in G1 medium at 37 °C in a 5% CO_2_ atmosphere with saturated humidity for 1 h. The reconstructed embryos were activated with a 6 h incubation in Ca^2+^-free CZB containing 10 mM SrCl_2_, 5 μg/ml CB and 5 nM Scriptaid. After a subsequent incubation in G1 containing 5 nM Scriptaid for 3 h, the reconstructed embryos were thoroughly cultured in G1 and G2 (after the 4-cell stage) medium.

### Embryo collection

**Fig. 1:**
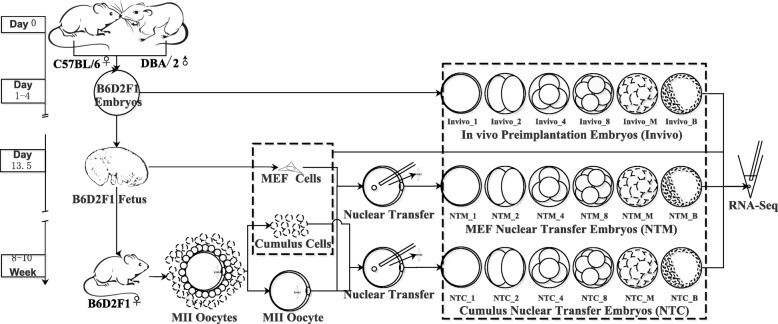
The derivation of embryos and cells with the same genetic background. Twenty different samples shown in the two dashed boxes were used for RNA-seq analyses. All samples were on BDF1 genetic background. The genomes of the F1 generation were obtained by crossing a C57BL/6 female mouse and a DBA/2 male mouse

As shown in Fig. 1, in vivo embryos (in vivo group) were collected from C57BL/6 female mice that had been mated with DBA2 male mice. These embryos were collected at the following six stages of development: pronuclear, 2-cell, 4-cell, 8-cell, morula and blastula stages (designated in vivo_1, in vivo_2, in vivo_4, in vivo_8, in vivo_M and in vivo_B, respectively). MEF donor NT embryos (NTM group) were reconstituted with BDF1 MEFs. Female C57 mice were mated with male DBA mice, and 13.5-day-old fetuses were obtained to produce the original MEFs. These MEFs with the BDF1 genetic background were sub-cultured, and cells at the 3rd-5th passages were used as donor cells. The MII oocyte cytoplasm was obtained from 8- to 10-week-old BDF1 female mice. Six stages of reconstituted NTM embryos were collected after different culture periods. NTM embryos from the pronuclear to blastula stages were designated NTM_1 to NTM_B. The CC donor NT embryos (NTC) were reconstituted with CCs collected from BDF1 female mice. The NTC embryos were collected in the same manner as the NTM embryos. NTC embryos from the pronuclear stage to the blastula stage were designated NTC_1 to NTC_B.

The study included 18 groups to represent the six stages of development in each of the three types of embryos. Three biological replicates from each group were prepared. Then, we used 54 embryos for the single-embryo RNA-seq. These 54 embryos were chosen from 904 embryos based on stringent morphological criteria.

### Single-embryo RNA-seq library generation

Each embryo was transferred to lysate buffer in a PCR tube and immediately stored at − 80 °C. A diluted ERCC mix (Life Technologies 4456740) was added to the lysis buffer as a spike-in for each sample. Reverse transcription was performed directly on the cytoplasmic lysate. Terminal deoxynucleotidyl transferase was then used to add a poly(A) tail to the 3′-end of the first-strand cDNAs. The total cDNA library of the single cells was then amplified for 18–20 cycles to construct the library. The amplified cDNA library was fragmented using a Covaris sonicator (Covaris S220, Woburn, MA, USA). The KAPA Hyper DNA Library Prep Kit (KK8504, Kapa Biosystems) was used according to the manufacturer’s instructions to generate the sequencing libraries. Paired-end 150-bp sequencing was further performed on a HiSeq 2500 (Illumina) platform at the Annoroad Gene Technology Corporation, Ltd. (Beijing, China).

### Analysis of the RNA-seq data

A Perl script was used to filter the original data (raw data) and guarantee the quality of the data for the analysis. The filtering step using the Perl script included the removal of adapters, redundant reads and low-quality reads. After the filtering process, the clean data were assessed for data quality, including Q30 statistics, data quantity statistics, and base content statistics. The reference gene and genome annotation files were downloaded from UCSC and used to build the reference genome library using Bowtie2 v2.2.3 software. Then, the clean data were mapped to the reference genome using TopHat v2.0.12 software. In addition, Bowtie2 software was used to map and compare the data obtained from TopHat to enhance the accuracy of the mapping results. IGV (Integrative Genomics Viewer) was used to view the mapped results in a heatmap, histogram, scatter plot and other formats. HTSeqv0.6.0 was used to calculate the number of copies of each gene, and FPKM was used to quantitatively assess expression. The significance of the differentially expressed genes was calculated using DESeq software by performing pair-wise comparisons among the different types of embryos. The *P*-value adjusted for multiple tests and absolute value of log2 fold change were obtained to determine whether the genes were significantly differentially expressed. Blast2go was used for the GO analysis, and KAAS was used for the KEGG analysis. DAVID bioinformatics resource version 6.8 was used for the GO enrichment analysis and protein interactions analysis (https://david.ncifcrf.gov/home.jsp). The significance of the differential GO terms was calculated using above mentioned software by performing pair-wise comparisons, and three independent examples were used in each groups. Heatmaps were generated using the “heatmap.2” function in R. Venn diagrams were generated using Venny2.1 on the website http://bioinfogp.cnb.csic.es/tools/venny/index.html.

### PCA analysis

PCA analyses were performed based on FPKM values using the “prcomp” function in R. The publicly available SCNT expression data were downloaded from the GEO repository (GSE70608). We first adjusted for the potential batch effect between the datasets using “ComBat” function in “sva” package to compare the GEO data with our data. Then, the batch-corrected data were subjected to a PCA clustering analysis. Similarities between the publicly available data and our data were evaluated by calculating Pearson’s correlation coefficients for the FPKM values of all genes.

## Results

### The derivation of in vivo embryos, NT embryos and donor cells with the same genetic background

Embryos and cells were collected using the methods shown in Fig. 1 to enable an exact syngeneic comparison of in vivo embryos and NT embryos. In vivo embryos were collected from C57BL/6 female mice that had been mated with DBA/2 male mice. NTM embryos were reconstructed with BDF1 MEF nuclei and the MII oocyte cytoplasm. The NTC embryos were reconstituted with CCs that were collected from BDF1 female mice. Therefore, the genetic background of all three types of embryos was BDF1. The donor cell CCs and MEFs were also prepared in three biological replicates for the single-embryo RNA-seq analysis. Sixty samples were analyzed.

### Transcriptional profiles of in vivo embryos, NT embryos and donor cells

Using the Illumina HiSeq2000 sequencer, we generated 387 Gb of clean sequencing data from 60 samples with an average of 43 million clean reads per sample and a read length of 150 bp. Compared to the mouse reference genome sequence, the mapping rate was 90–96% (an average of 94.5%). This information is recapitulated in the boxplot shown in Additional file 1: Figure S1. In total, 26845 genes (FPKM in Additional file 2: Table S3) were detected in the 60 samples, accounting for 57% of the known genes in mice. Among the 54 embryo samples, 25943 genes were detected, accounting for 55% of the known genes in mice. Therefore, the mouse preimplantation embryo expresses more than half of the known genes in the mouse.

**Fig. 2:**
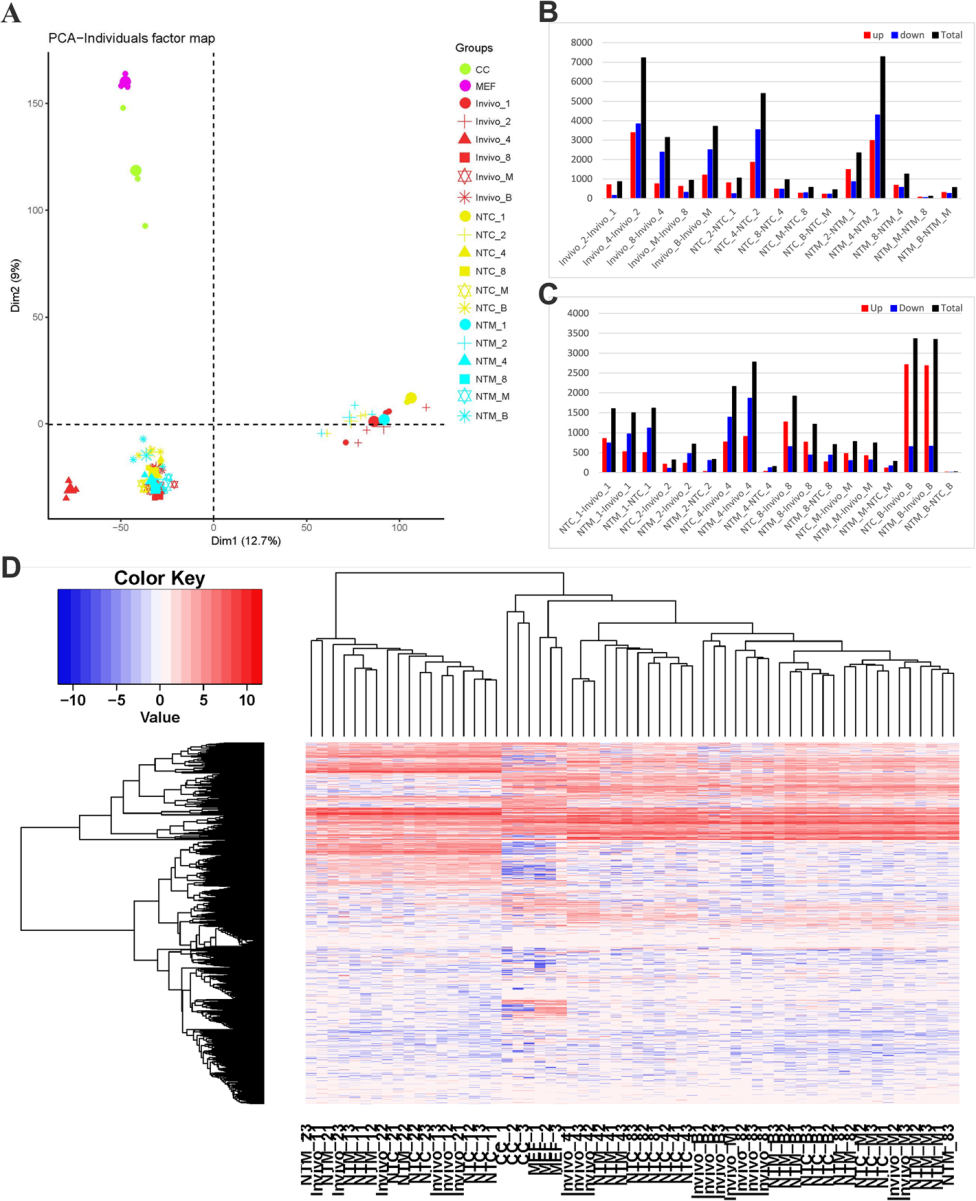
Single-cell transcriptional profiles of in vivo embryos, NT embryos and donor cells. **a** PCA of all 60 samples revealing key relationships between groups. Smaller symbols represent each sample. Bigger symbols represent the average of samples from each group. **b** The number of genes displaying differential expression between adjacent developmental stages. **c** The number of genes exhibiting differential expression between different types of embryo at the same stage. **d** Heatmap of all 60 samples. Blue indicates lower expression and red indicates higher expression. The hierarchical clustering analysis of the samples is shown on top of the heatmap. The hierarchical clustering analysis of the genes is shown on the left. NTM_23 means the third sample of 2-cell NTM embryos. CC_1 means the first sample of cumulus cells, etc.

A principal component analysis (PCA) was performed to examine the obtained data (Fig. 2a). The two types of donor cells were clustered together and segregated from the other samples, conforming to the biological laws of cell differentiation. The mouse ZGA occurred at the 2-cell stage. The transcription patterns were obviously different between the 1–2 cell stage and subsequent stages. Our PCA findings are consistent with these data. The three types of 1-cell embryos and 2-cell embryos clustered together, but the three types of 4-cell, 8-cell, morula and blastula embryos were clustered in another region.

The gene transcription patterns were significantly changed during embryonic development (Fig. 2b). The major ZGA stage occurs in the late 2-cell embryos in mouse, which corresponds to our 2–4 cell stage embryos. The largest transcriptional difference appeared in this stage, as the transcription of approximately 6000 genes exhibited changes. Significantly fewer up-regulated genes were observed in both NT embryos than in the in vivo embryos, indicating that many genes failed to be activated in both NT groups during the major ZGA stage. The lack of these transcripts may be a reason for the poor development of NT embryos. During the morula-blastula period, more than 3,000 transcripts were altered in the in vivo group, but only approximately 500 differences were observed in both NT groups. Therefore, the in vivo group displays greater alterations in transcription than the NT groups (*P* < 0.05). The first cell differentiation step occurred during this period. These erroneous transcription steps may affect the differentiation of trophoblastic cells, which may further cause the placental abnormality after NT embryo implantation.

Many genes are differentially expressed between NT embryos and in vivo embryos (Fig. 2c, Additional file 3: Table S4). The greatest difference was observed between the blastula stage and 4-cell stage. In the blastula stage, more than 3,000 genes were differentially expressed between NT groups and the in vivo group. More than 70% of these genes were up-regulated in NT groups compared with the in vivo group. In the 4-cell stage, approximately 2500 genes were differentially expressed between the NT groups and the in vivo group. Approximately 1/3 of these genes were down-regulated in NT groups, a potential cause of the poor ZGA in the NT embryos.

The maximum differences in transcription (1618 genes) observed between the two types of NT embryos were detected at the 1-cell stage (Additional file 4: Figure S2A). This stage is the initial step in cellular reprogramming. Donor cells (CCs or MEFs) had just been injected into the cytoplasm. The functions of 1618 genes were analyzed using the GO method. Nucleus (GO: 0005634) was the term displaying the greatest difference (*P* = 2.9E-42). Thus, the transcriptional differences between 1-cell NTC and NTM embryos were more likely due to the use of different donor cells. The term in the Biological Process category exhibiting the greatest difference was Transcription, DNA-templated (GO: 0006351, *P* = 4.7E-11). Regulation of transcription, DNA-templated (GO: 0006355, *P* = 1.6E-10) was also obviously different. Based on these findings, the cellular reprogramming was not complete and substantial differences were observed between two NT groups. Transcriptional co-differences between NTC VS. Invivo and NTM VS. Invivo were shown in Additional file 4: Figure S2B. only 402 genes co-difference in the 1-cell stage. The differences observed in the 1-cell stage were substantially decreased (less than 1/4 remained) when the NT embryos developed further. Thus, most reprogramming events occurred in the 1-cell stage. The minimum differences (only 20 genes) between the two types of NT embryos were detected in the blastula stage (Additional file 4: Figure S2A). Therefore, based on the lack of significant differences between the two types of NT embryos, SCNT should have the ability to reprogram different donor cells to the same status. However, reprogramming to the same status does not represent the normal developmental status. More than 3000 genes were differentially expressed between NT groups and the in vivo group. Therefore, the transcriptional defects observed in NT embryos did not depend on the type of donor cells.

According to the heatmap of all the 60 samples (Fig. 2d), the transcriptional patterns in donor cells (CCs and MEFs) was distinct from the various embryos. This finding is consistent with the PCA results. According to the hierarchical clustering analysis (Fig. 2d), pronuclear and 2-cell stage in vivo and NT embryos exhibited similar differences in transcription and clustered together. The embryos in 4-cell, 8-cell, morula and blastula stages were clustered together on the other side. Thus, significant changes in transcription occurred between the 2-cell and 4-cell stages.54 embryonic samples were used for weighted gene co-expression network analysis. Gene clustering is performed based on the software WGCNA and the correlation between the two genes. 60 modules with the same expression pattern were marked with different color markers (Additional file 5: Figure S5A). By calculating the correlation between these modules and embryonic characteristics, the influence relationship between modules and embryonic characteristics were obtained (Additional file 5: Figure S5B). Eigengene adjacency heatmap were used to cluster modules with embryonic characteristics, where the redder the color, the greater the correlation between the two (Additional file 5: Figure S5C). Five modules are strongly correlated with developmental stage. Three of them are positively correlated: Lightcyan (*r* = 0.8, *P* = 4E-12), Sienna3 (*r* = 0.7, *P* = 8E-8), Coral1 (*r* = 0.5, P = 8E-5). Two of them are negative correlation: Turquoise (*r* = − 0.8, P = 4E-16), Darkseagreen4 (*r* = − 0.6, *P* = 1E-5). What is particularly noteworthy is that no one module is strongly correlated with the type of donor cells. The genes of Lightcyan, Sienna3 and Turquoise were analyzed by the GO method (Additional file 5: Figure S5D). The other two modules unable to analyze by the GO method for the insufficient genes. According to the GO analysis, most significant terms is belong to cellular component (CC) group. Cell cycle (GO: 0007049) and cell division (GO: 0051301) are the most significant terms of biological process group in the negative correlation modules. Additional enrichment analysis in Sienna3 module shows that ribosome related term is the most significant group (Enrichment Score 10.87, detail in Additional file 5: Figure S5E). All these WGCNA data suggest that the NT embryos is similar to the in vivo embryos during development. NT embryos produced by different donor cells showed little difference.

### A deficiency in translation initiation is the main defect in 1–2 cell NT embryos

**Fig. 3:**
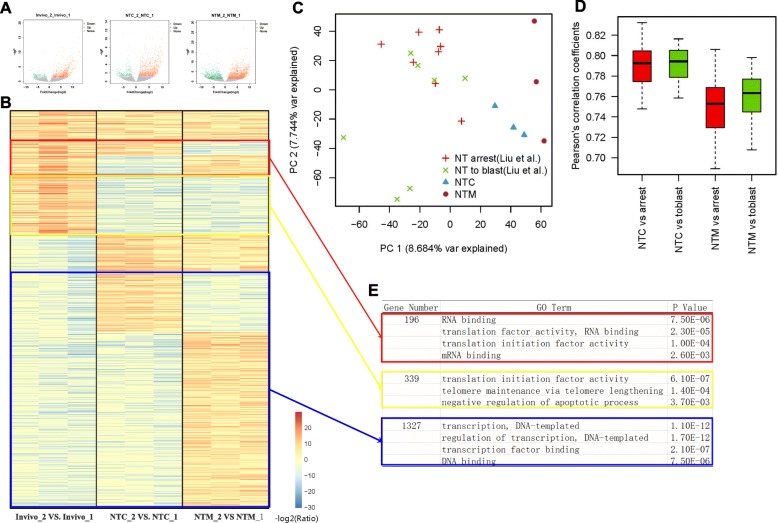
Analyses of differentially expressed genes in 1–2 cell stage embryos. **a** Volcano plot of 1–2 cell stage embryos. Green dots indicate down-regulated genes in the 2-cell stage compared with the 1-cell stage, red dots indicates up-regulated genes in the 2-cell stage compared with the 1-cell stage, and gray dots indicates genes that did not display a difference in transcription between the 2-cell and 1-cell stages. Every plots' Ensembl genes ID were listed in Additional file 7: Table S2. **b** Heatmap of all up-regulated genes (red dots) and down-regulated genes (green dots) shown in (**a**). Blue indicates lower expression and red indicates higher expression. Three clustered genes were selected with square. Clustered genes in the red square were expressed at higher levels in the in vivo group but displayed higher expression in only one of the NT groups. Clusters displaying similar changes are depicted by the yellow square and blue square. **c** PCA analysis of our data compared to GSE70608 in 2-cell NT embryos. Red crisscross (NT arrest): 2-cell embryos are developmentally arrested in the 2-cell stage (GSE70608). Green multiplication sign (NT to blast): 2-cell embryos develop to the blastula stage (GSE70608). Blue triangle: Our 2-cell NTC embryos. Purple circle: Our 2-cell NTM embryos. **d** Correlation analysis of our data compared to GSE70608 in 2-cell NT embryos. **e** GO analyses of clustered genes selected as described in (**b**)

The transcription of certain genes was altered during the 1–2 cell stage (minor ZGA), as shown in Fig. 3. Specific differences in each group are shown in a volcano plot (Fig. 3a). We generated a heatmap of the genes that were significantly up-regulated or down-regulated, as shown in Fig. 3b. Of the 704 genes that were up-regulated in the in vivo group, only 169 genes were up-regulated in both NT groups. More importantly, nearly half of these genes (339 genes, Additional file 6: Table S1) were down-regulated in both NT groups. A discussion of the in-depth analysis of the 339 genes is provided below. Of the 1,552 genes that were down-regulated in the in vivo group, only 8 genes (0.5%) were also down-regulated in the two NT groups. Most down-regulated genes exhibited a completely different pattern in the two NT embryos, which may be due to the different nuclear sources of these embryos. CCs, MEFs and zygotes likely silence different genes during this period. Are these differences due to reprogramming or a poor NT procedure? We compared our data with the existing dataset GSE70608 [13]. According to the PCA analysis (Fig. 3c) and correlation analysis (Fig. 3d), our 2-cell samples exhibited good consistency and can be considered perfectly reprogrammed samples.

Gene ontology (GO) analyses of these genes are shown in Fig. 3c. Three hundred thirty-nine oppositely regulated genes were enriched in the translation initiation factor activity term (GO: 0003743), including well-known genes, such as Eif1ad, Eif3j2, and Eif1ax. According to a SMART term analysis, the protein domain that is most frequently observed in these genes is the eukaryotic translation initiation factor A (eIF-4C) (*P* = 1.6E-3). EIF-1A is a protein required for the maximal rate of protein biosynthesis. EIF-1A enhances ribosome dissociation into subunits and stabilizes the binding of the initiator Met-tRNA to the 40S ribosomal subunits [30]. According to an INTACT analysis, these genes interact with upstream binding transcription factors, such as RNA polymerase I, Nanog and Rasl11a. The 339 oppositely regulated genes in both NT groups were strongly correlated with translation initiation. The lack of translation initiation activity may result in a deficiency in key factors needed during the main ZGA period, leading to additional deficiencies during NT embryo development.

### A lack of RNA processing and modification procedures are the main defects in 2–4 cell NT embryos

**Fig. 4:**
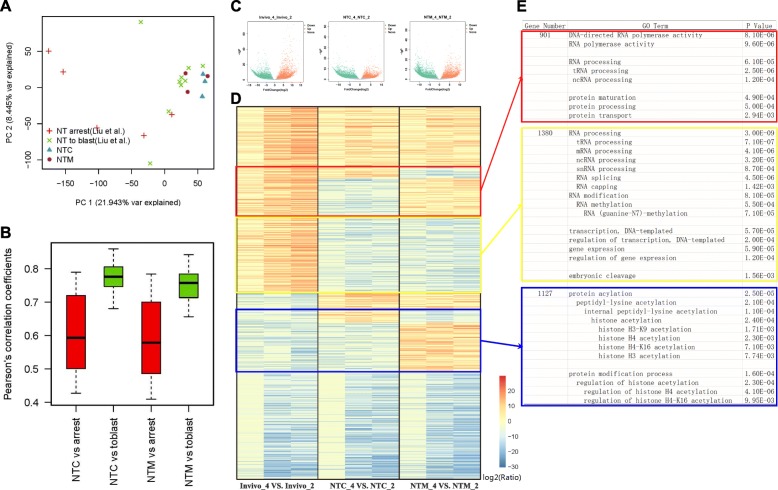
Analyses of differentially expressed genes in 2–4 cell stage embryos. **a** PCA analysis of our data compared to GSE70608 in 4-cell NT embryos. Red crisscross (NT arrest): 4-cell embryos are developmentally arrested in the 4-cell stage (GSE70608). Green multiplication sign (NT to blast): 4-cell embryos develop to the blastula stage (GSE70608). Blue triangle: Our 4-cell NTC embryos. Purple circle: Our 4-cell NTM embryos. **b** Correlation analysis of our data compared to GSE70608 in 4-cell NT embryos. **c** Volcano plot of 2–4 cell stage embryos. Green dots indicate down-regulated genes in the 4-cell stage compared with the 2-cell stage, red dots indicate up-regulated genes in the 4-cell stage compared with the 2-cell stage, and gray dots indicate genes that did not display differences in transcription between the 4-cell and 2-cell stages. Every plots' Ensembl genes ID were listed in Additional file 7: Table S2. **d** Heatmap of all up-regulated genes (red dots) and down-regulated genes (green dots) shown in (**a**). Blue indicates lower expression, and red indicates higher expression. Three clustered genes were selected with a square. The meanings of the clusters are explained in Fig. 3. **e** GO analyses of clustered genes selected as described in (**b**)

We compared our data with the existing dataset GSE70608 to determine the quality of the data obtained from the NT 4-cell embryos [13]. As shown in Fig. 4a, our samples correlated with each other and more closely resembled the samples that developed to blastulas. We also performed a correlation analysis (Fig. 4b). The correlation coefficient of our samples VS. NT to blast samples was greater than the arrested samples. Thus, our 4-cell samples displayed good consistency and can be considered perfectly reprogrammed samples.

During the major ZGA period, embryos must complete the activation of most zygote genes and gradually replace the maternal factors with the zygotic genes. An overview of the differentially expressed genes in each group is shown in a volcano plot (Fig. 4c). A heatmap was constructed with the genes displaying significant differences in expression, as shown in Fig. 4c. According to Fig. 4d, among the 3385 up-regulated genes in the in vivo group, approximately 1/3 (1104) of the genes in both the NT groups were transcribed correctly (Fig. 4d). The proportion of correctly transcribed genes was higher than during the 1–2 cell stage (*P* < 0.05). In both NT groups, 1380 genes (1/3) were oppositely regulated, a value that was also less than the 1–2 cell period (1/2) (P < 0.05). In summary, the differences in transcription between the NT groups and the in vivo group were significantly reduced during the 2–4 cell period, which was characterized by significantly fewer differences in transcription than the 1–2 cell period. Moreover, the differences in transcription between the NTC group and NTM group at this stage were significantly less than those observed during the 1–2 cell period.

The GO analysis of these differentially expressed genes is shown in Fig. 4e. In both NT groups, 1380 genes were oppositely regulated. The most enriched GO terms are related to RNA processing and modification. The lack of these processes causes problems in many correlated terms, such as gene transcriptional expression terms and embryo cleavage term. According to a Kyoto Encyclopedia of Genes and Genomes (KEGG) analysis of these 1380 genes, the spliceosome pathway (Additional file 8: Figure S3) showed remarkable abnormalities. During mRNA precursor splicing, introns are excised, and exons are joined by the spliceosome. The standard spliceosome consists of five small nuclear ribonucleoproteins, namely, U1, U2, U4, U5, and U6 snRNPs, and several spliceosome-associated proteins. All five snRNP genes exhibited serious errors during transcription in the NT groups (Additional file 8: Figure S3), potentially resulting in serious errors during the assembly of the mRNA precursor.

In addition to the oppositely regulated genes in both NT groups, many oppositely regulated genes were present in one NT group (Fig. 4d). Among the up-regulated genes in the in vivo group, 901 oppositely regulated genes were observed in one NT group. Most of these 901 genes were clustered in the GO terms RNA polymerase activity, RNA processing and protein processing (Fig. 4e). During the main ZGA period, numerous zygotic genes must be transcribed by RNA polymerase and mRNAs must be processed. The proteins translated during the minor ZGA period also require substantial protein processing to form mature proteins. According to our results, these processes were enhanced in the in vivo group but were lost in the NTC and NTM groups. Among the down-regulated genes in the in vivo group, 1127 oppositely regulated genes were observed in the single NT group. These genes were mainly clustered in histone acetylation and its regulatory GO terms (Fig. 4e). Recently, many histone modifications have been identified as reprogramming barriers, including H3K9me3, H3K4me3, and H3K27me3. According to our results, the in vivo embryos exhibited reduced acetylation at many histone sites during the 2–4 cell period, but the NT embryos did not display reduced acetylation. The acetylation of histone H3 or H4 may also be a potential reprogramming barrier according to the GO analysis.

Overall, we concluded that in vivo embryos exhibit enhanced RNA transcription and processing activities, an enhanced protein processing capacity, reduced histone acetylation, and enhanced post-translational modifications to generate greater numbers of correct mature RNAs and proteins for a proper ZGA in mouse embryos. However, NT embryos have not properly regulated these abilities, particularly the RNA processing and RNA modification activities. These defects may be among the most important explanations for the poor developmental ability of NT embryos.

### The kinase activity required for protein phosphorylation is not properly reduced during the NT morula-blastula stage, causing abnormally high gene transcription in blastulas

**Fig. 5:**
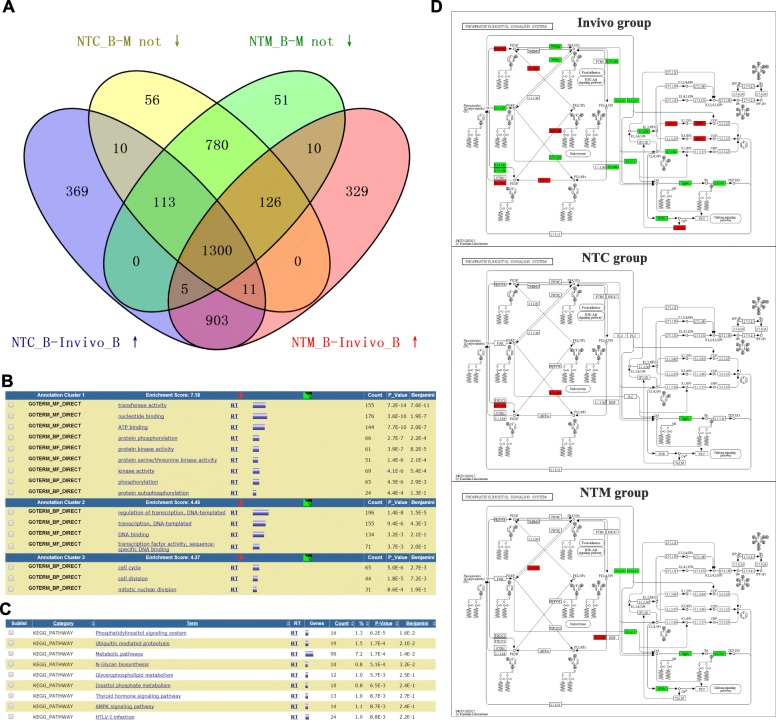
Analyses of differentially expressed genes in morula-blastula-stage embryos. **a** Venn diagram showing four groups of genes: yellow, genes that were not down-regulated in the NTC group compared to the in vivo group during the morula-blastula period; green, genes that were not down-regulated in the NTM group compared to the in vivo group during the morula-blastula period; blue, genes expressed at higher levels in the NTC blastula than in the in vivo blastula; and red, genes expressed at higher levels in the NTM blastula than in the in vivo blastula. **b** GO analyses of 1300 key genes selected as described in A. **c** KEGG analyses of 1300 key genes selected as described in (**a**). **d** Detailed description of the differences in the phosphatidylinositol signaling pathway. Red indicates up-regulated transcription when the morula developed to the blastula in each group, whereas blue indicates down-regulated transcription

Based on the analysis shown in Fig. 2b, we posed the following question: Are these different transcription patterns in the NT blastulas caused by transcription defects during the morula-blastula stage? We constructed a Venn diagram, as shown in Fig. 5a. Among the 2396 genes that had not been properly down-regulated during the development of morula-stage NTC embryos to the blastula stage, 1434 (59.85%) genes maintained an abnormally high transcription level in the NTC blastula. Among the 2385 genes that had not been properly down-regulated during the development of morula-stage NTM embryos to the blastula stage, 1441 (60.42%) genes maintained abnormally high transcription levels in the NTM blastula. One thousand three hundred of these genes (1434 and 1441) were identical. These 1300 genes (listed in Additional file 6: Table S1) accounted for 58.58% of all genes displaying abnormally high transcription (2219) in both NT blastocysts. In conclusion, most genes that exhibited abnormally high transcription in both NT blastula-stage embryos are due to defective down-regulation during development of the morula to the blastula.

We performed a GO enrichment analysis to further study the functions of these 1300 genes. According to the enrichment analysis, 73 clusters were identified. In the main cluster, 276 genes were enriched in 9 GO terms (Fig. 5b). These GO terms were related to kinase activity during the protein phosphorylation process. These proteins include Tgfbr1, which physically interacts with 8 proteins (Additional file 9: Figure S4A) that are related to embryonic growth retardation and embryonic lethality during organogenesis [31], and kif3a, which physically interacts with Kif3bK, if3c and Tnks to induce abnormal embryonic development [32]. In the second enriched cluster, 247 genes were associated with 4 GO terms. These terms were associated with transcription factor activity in the DNA binding term. The protein-protein interactions of these genes were analyzed (Additional file 9: Figure S4B). Nine proteins interacted with 4 to 18 separate proteins (*P* < 0.01). The most significantly enriched protein was the transcription factor CP2-like 1 (Tfcp2l1), which is an Oct4-interacting protein [33]. Tfcp2l1 not only induces the reprogramming of the primed state of pluripotent ESCs into the naive state but is also sufficient to maintain the naive pluripotent state in ESCs [34]. One hundred thirteen relationships were observed among Tfcp2l1-, Oct4- and Oct20-related genes (Additional file 9: Figure S4C). Two of these 20 genes are included in the 1300 genes. One of these genes, i.e., calcineurin binding protein 1 (Cabin1), also belongs to the enzyme regulator GO term. The knockout of Cabin1 results in embryonic lethality during organogenesis [35]. In the third enriched cluster, 67 genes were associated with 3 GO terms. These terms are associated with the cell cycle and cell division. These genes influence the cell cycle signaling pathway (*P* = 3.5E-7). Among these 67 genes, 8 of the encoded proteins interacted with S_TKc, the catalytic domain of serine/threonine protein kinases.

We performed a KEGG enrichment analysis of these genes to further investigate the mechanisms by which these 1300 genes perform their functions. The results are shown in Fig. 5c. Nine signaling pathways were significantly affected by these genes. Most pathways were related to phospholipid metabolism, such as the phosphatidylinositol signaling system (*P* = 6.2E-5, Fig. 5d) and inositol phosphate metabolism (*P* = 6.5E-3). These results are consistent with the results from our GO analysis, further confirming that these genes mainly utilize the phospholipid signaling pathway to affect the activity of kinases required for protein phosphorylation.

### Two thousand three constant genes in donor cells are not reprogrammed during the development of NT embryos

**Fig. 6:**
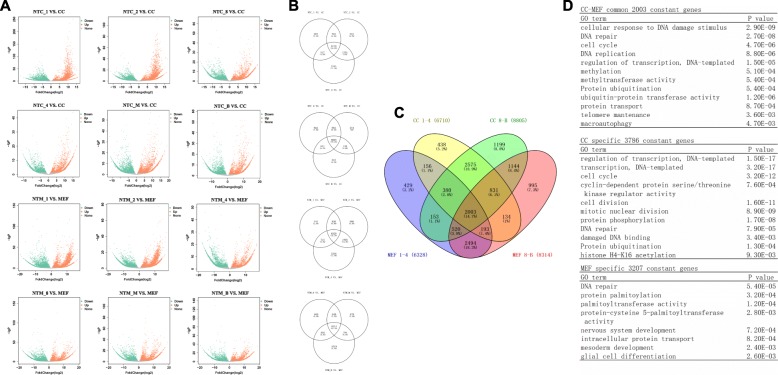
Analyses of donor cell genes that maintain constant transcript levels during embryonic development. **a** Volcano plot of NT embryos (NTC or NTM, 6 stages) VS. corresponding donor cells (CCs or MEFs). Every plots' Ensembl genes ID were listed in Additional file 7: Table S2. **b** Venn diagram of constant genes (gray dots) in the volcano plot in the same line. **c** Venn diagram of the shared constant genes from each Venn diagram shown in (**b**). **d** GO analysis of key constant genes in each group shown in (**c**)

The memory of the somatic cell identities inhibits the reprogramming and development of NT embryos. RNA-seq detected 14405 genes in the CCs and 15379 genes in the MEFs. After nuclear reprogramming, some genes were up-regulated or down-regulated at different stages of NT embryonic development. In each stage, many genes did not display changes in transcription (gray dot in Fig. 6a). A Venn diagram of these genes was constructed for each group (Fig. 6b). According to an analysis of the Venn diagram, 5789 (CCs) and 5210 (MEFs) genes did not display changes in transcription at each embryonic stage (Fig. 6c). These genes were designated constant genes. Of these constant genes, 2003 genes (listed in Additional file 6: Table S1) were the same in both CCs and MEFs (Fig. 6c). According to the GO analyses, these 2003 genes are mainly related to DNA damage repair (Fig. 6d). The 3,786 CC-specific genes were mainly enriched in the GO terms related to cell transcriptional regulation and cell division. The 3207 MEF-specific genes were mainly enriched in the GO terms MEF differentiation and development.

Even in normal embryos, some genes may be expressed at constant levels; therefore, these constant genes are not necessarily barriers. In the next section, we compared the transcript levels of these constant genes in the NT embryos with the in vivo embryos during each embryonic stage to screen for potential reprogramming barrier genes.

### The 136 potential reprogramming barrier genes are mainly related to the positive regulation and production or secretion of certain factors

**Fig. 7:**
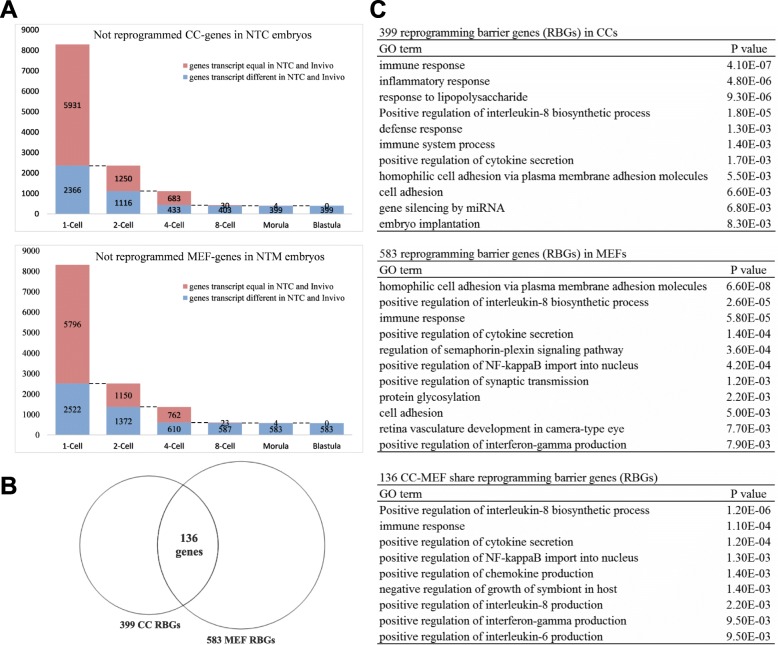
Analyses of reprogramming barrier genes (RBGs) in donor cells. **a** CC/MEF-specific genes that were not reprogrammed during NTC/NTM embryonic development. The genes analyzed in the next column were obtained from the “blue area” in the previous column. **c** Venn diagram of shared RBGs shown in (**a**). **d** GO analysis of RBGs selected from (**a** and **b**)

Three hundred ninety-nine genes were regarded as potential reprogramming barrier genes (RBGs) in CCs (Fig. 7a). Five hundred eighty-three potential RBGs were identified among the 16735 genes in MEFs. These potential RBGs were neither reprogrammed nor correctly transcribed during embryonic development. These potential RBGs must be the key genes responsible for improving the somatic reprogramming efficiency.

More than half of these potential RBGs are not present in the existing GO database, suggesting that this group of genes has not been well studied. We analyzed the annotated potential RBGs, as shown in Fig. 7. The potential RBGs in CCs were mainly enriched in the GO terms immune defense and regulating cytokines. Significant effects on genes related to miRNA silencing and embryo implantation were observed (Fig. 7c). The potential RBGs in MEFs were mainly enriched in the GO terms cell adhesion, regulating cytokines, and immune response. These genes have a significant influence on neural development and related protein expression.

Furthermore, 136 key genes (listed in Additional file 6: Table S1) were present among the 399 CCs potential RBGs and 583 MEF potential RBGs (Fig. 7b). The GO terms of these 136 genes were enriched in more specific processes (Fig. 7c), including the positive regulation of the production or secretion of interleukin, cytokines, NF-kappa B, chemokines and interferon-gamma. Therefore, the genes encoding proteins that regulate the production and secretion of various factors maybe not reprogrammed correctly. These 136 differential genes may be reprogramming barrier genes (RBGs) and more studies are needed to identify.

## Discussion

In this study, we obtained the transcriptome blueprints of all stages of NTC embryos, NTM embryos and in vivo embryos using single-embryo RNA-seq technology. NT embryos exhibit translation initiation defects during the minor ZGA period and RNA processing and RNA modification defects during the major ZGA period as well as impaired activity of the protein kinases required for protein phosphorylation during blastocyst formation. The discovery of these defects in NT embryos will assist researchers developing future studies to explore the mechanism of development in NT embryos and further improve NT efficiency, thus promoting the early and broad application of the technology. In the present study, we also proposed for the first time that the barrier to somatic nuclear reprogramming includes 136 RBGs among 2003 constant genes (previously screened) that are primarily related to the positive regulation of various factors. Histone H4K16 acetylation is a potential somatic reprogramming barrier. These findings reveal candidate barriers to somatic nuclear reprogramming and provide new ideas for in-depth studies designed to explore the mechanism of nuclear reprogramming and improve the efficiency of nuclear reprogramming.

Many existing defects in NT embryos were obtained through somatic reprogramming, such as blocks to early embryonic development and an abnormal placenta. Blocks in mouse embryonic development occurred during the 2-cell stage. The major ZGA period occurs during this stage. Many proteins have been shown to play a decisive role in the ZGA [36]. Nuclear TCA proteins and metabolites are essential for the human and mouse ZGA [37]. A new reprogramming factor, i.e., BRD3R, promotes reprogramming by regulating mitosis in early embryos [38]. the ZGA requires the transcription factors Zelda (*Drosophila*) [39] and DUX (mouse) [40]. If the processing of these proteins is affected, the ZGA might be seriously disturbed and block the development of the embryo. DUX (ENSMUSG00000075046), Nanog (ENSMUSG00000012396) and some other genes exhibited abnormal transcription in the NT embryos during the minor ZGA period (listed in Additional file 6: Table S1). Knock out models of certain genes on our list should validate more key genes. The overexpression of certain genes in NT pronuclear embryos and a subsequent examination of their effects on embryonic development is another method that should be exploited. Researchers should start with the transcription factors in the list. Our results also support the hypothesis that protein translation initiation is more critical during the minor ZGA period in mouse NT embryos. In the subsequent major ZGA period, RNA modification and processing are critical steps. Thus, improvements in translation initiation, RNA processing and RNA modification in early NT embryos may reduce the development block of early NT embryos. Placental enlargement is another common abnormality observed after mouse SCNT [41, 42]. The loss of placenta-specific gene imprinting and the down-regulation of certain genes have been observed in mice [43, 44] and cattle [45]. Trophoblast cells are the primary source of these placental defects [46, 47]. Abnormal miRNA expression in trophoblasts leads to abnormal placental development following SCNT [48]. In the present study, we observed additional abnormal transcripts during trophoblast differentiation, which provides a new strategy to further solve the problems of the SCNT-induced placental abnormality. To the best of our knowledge, this paper is the first to study the transcriptomes in mouse NT embryos at all stages using single-embryo RNA-seq. This systematic study allowed us to adequately reveal the transcriptional defects in NT embryos. In conclusion, the transcriptional defects identified in this study provide new strategies to solve the development abnormalities in the NT embryo.

The reprogramming barrier is another hot topic in recent reprogramming studies. Many epigenetic sites are considered reprogramming barriers, such as H3K9me3 [13, 29, 49–51], H3K4me2 [52], H3K4me3 [12, 13, 53, 54], H3K27me3 [49], H3K36me3 [51], DNA methylation [49, 55], and H2AK119 ubiquitination [49]. In the present study, H4K16 acetylation was postulated to be a reprogramming barrier in CC donor mouse NT embryos but not in MEF donor mouse NT embryos. Thus, even in the same species or genetic background, the reprogramming barriers in different types of somatic cells are not exactly the same. These epigenetic reprogramming barriers, in fact, regulate a group comprising multiple genes [49]. Due to the advent of single-embryo RNA-seq, a group of reprogramming barrier genes (RBGs) has been identified in *Xenopus* [49, 53] and cattle [56]. Changes in the transcription of this group of genes effectively improve the reprogramming efficiency [53, 56]. We selected 399 RBGs in CC cells and 583 RBGs in MEF cells by single-embryo RNA-seq. Of these genes, 136 identical RBGs were found in the CC RBGs and MEF RBGs, which may be more suitable representatives of mouse RBGs. Overexpression and knockdown/out are conventional methods used to discover gene function. The overexpression of kdm4d [29], kdm4b [13, 51], and kdm4a [50] alters the H3K9me3 pattern and improves the reprogramming efficiency. The overexpression of Kdm5b [13] alters the H3K4me3 pattern and also improves the reprogramming efficiency. The knockout of Dnmt1s [57] and Dnmt3l [58] in donor cells also improve the reprogramming efficiency. Thus, changes in the transcription of specific genes can improve the reprogramming efficiency [14]. In future studies, we aim to knockout certain RBG genes (listed in Additional file 6: Table S1) in CCs or MEFs, perform nuclear transfer with these somatic cells and then test the NT embryo development rate. Improvements in the NT embryonic development rate will further validate the effects of selected key RBGs and help to establish a new method for improving the efficiency of nuclear reprogramming in mice. In conclusion, we identified new potential epigenetic and transcriptional barriers in mouse somatic reprogramming and provided suggestions for several new strategies to improve the efficiency of somatic reprogramming.

## Conclusions

Altogether, our data not only provided a map of the transcriptome in all embryonic stages but also identified new transcription defects and the reprogramming barrier genes in mouse somatic cell reprogramming. Further investigations based on these results might enhance the early application of reprogramming technology in additional fields.

## Additional files

Additional file 1: Gene expression in each sample. (PDF 220 kb)

Additional file 2: FPKM values of every samples. All the genes' Ensembl gene ID and FPKM value of 60 samples were listed. (XLS 20764 kb)

Additional file 3: List of different genes between NT groups and Invivo group. Two group Ensembl gene IDs were listed. One is different genes between NTC embryos and Invivo embryos. The other is different genes between NTM embryos and Invivo embryos. (XLSX 182 kb)

Additional file 4: Analysis of transcription in NTM and NTC embryos. (PDF 209 kb)

Additional file 5: Ensembl gene IDs of selected cluster genes. (PDF 1632 kb)

Additional file 6: Ensembl gene IDs of selected cluster genes. Ensembl gene IDs were listed in the four columns. (XLSX 52 kb)

Additional file 7: Volcano plots in Fig 3-6. Ensembl gene IDs of each volcano plots in Fig 3-6 were listed. (XLSX 133 kb)

Additional file 8: Spliceosome KEGG pathway in the in vivo, NTC and NTM groups. (PDF 231 kb)

Additional file 9: Analysis of specific protein-protein interactions. (PDF 748 kb)
