# Supplementary material for: Transcriptional defects and reprogramming barriers in somatic cell nuclear reprogramming as revealed by single-embryo RNA sequencing
Source: BMC Genomics. 2018 Oct 10;19:734. doi: 10.1186/s12864-018-5091-1 (PMC6180508; doi:10.1186/s12864-018-5091-1)
Supplement: Supplementary file 4 — Analysis of transcription in NTM and NTC embryos. (PDF 209 kb) [file 12864_2018_5091_MOESM4_ESM.pdf]

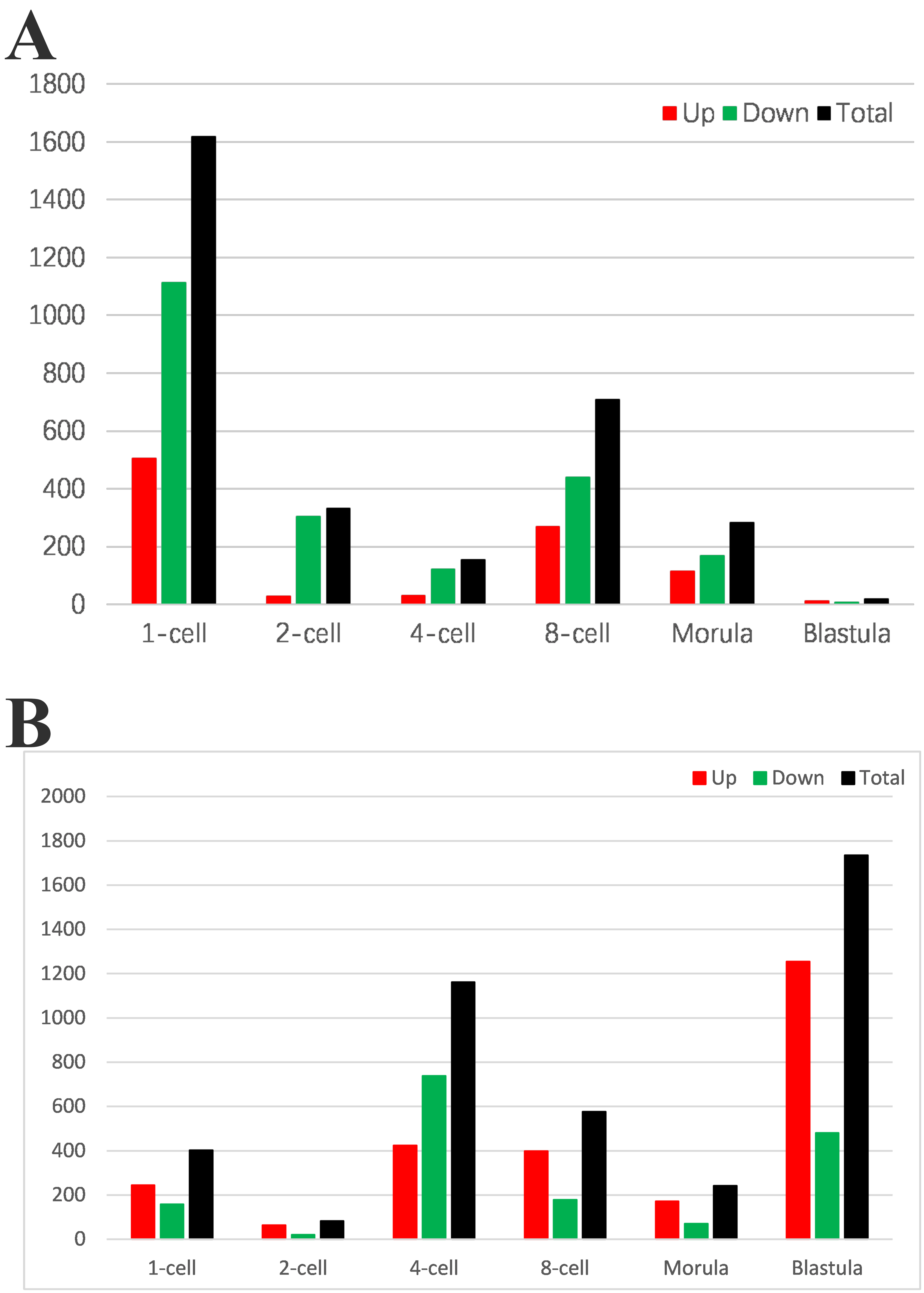

Figure S2 Transcriptional analysis of NTM and NTC embryos, Related to Figure 2.

(A) Transcriptional differences between NTM and NTC embryos. Red bar means the transcription is higher in NTM than in NTC embryos. Green bar means lower. Black bar means the total transcriptional difference between NTM and NTC.

(B) Transcriptional co-differences between NTC VS. Invivo and NTM VS. Invivo. Red bar means the quantity of genes which were higher transcript in both NT group than in Invivo group. Green bar means lower transcript. Black bar means the total transcriptional difference between both NT embryos and Invivo embryos.
