# Supplementary material for: Transcriptional defects and reprogramming barriers in somatic cell nuclear reprogramming as revealed by single-embryo RNA sequencing
Source: BMC Genomics. 2018 Oct 10;19:734. doi: 10.1186/s12864-018-5091-1 (PMC6180508; doi:10.1186/s12864-018-5091-1)
Supplement: Supplementary file 9 — Analysis of specific protein-protein interactions. (PDF 748 kb) [file 12864_2018_5091_MOESM9_ESM.pdf]

A

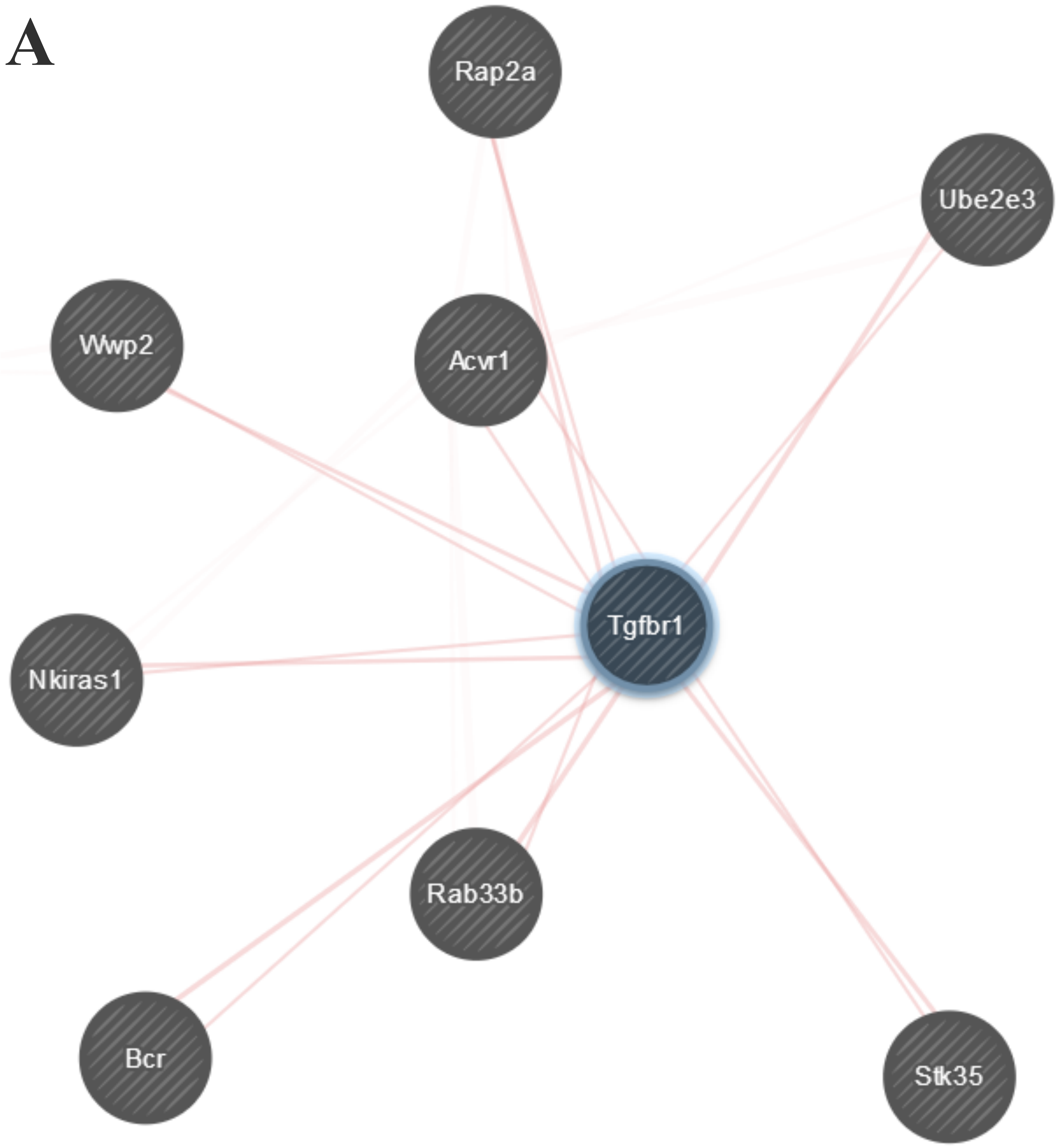

C

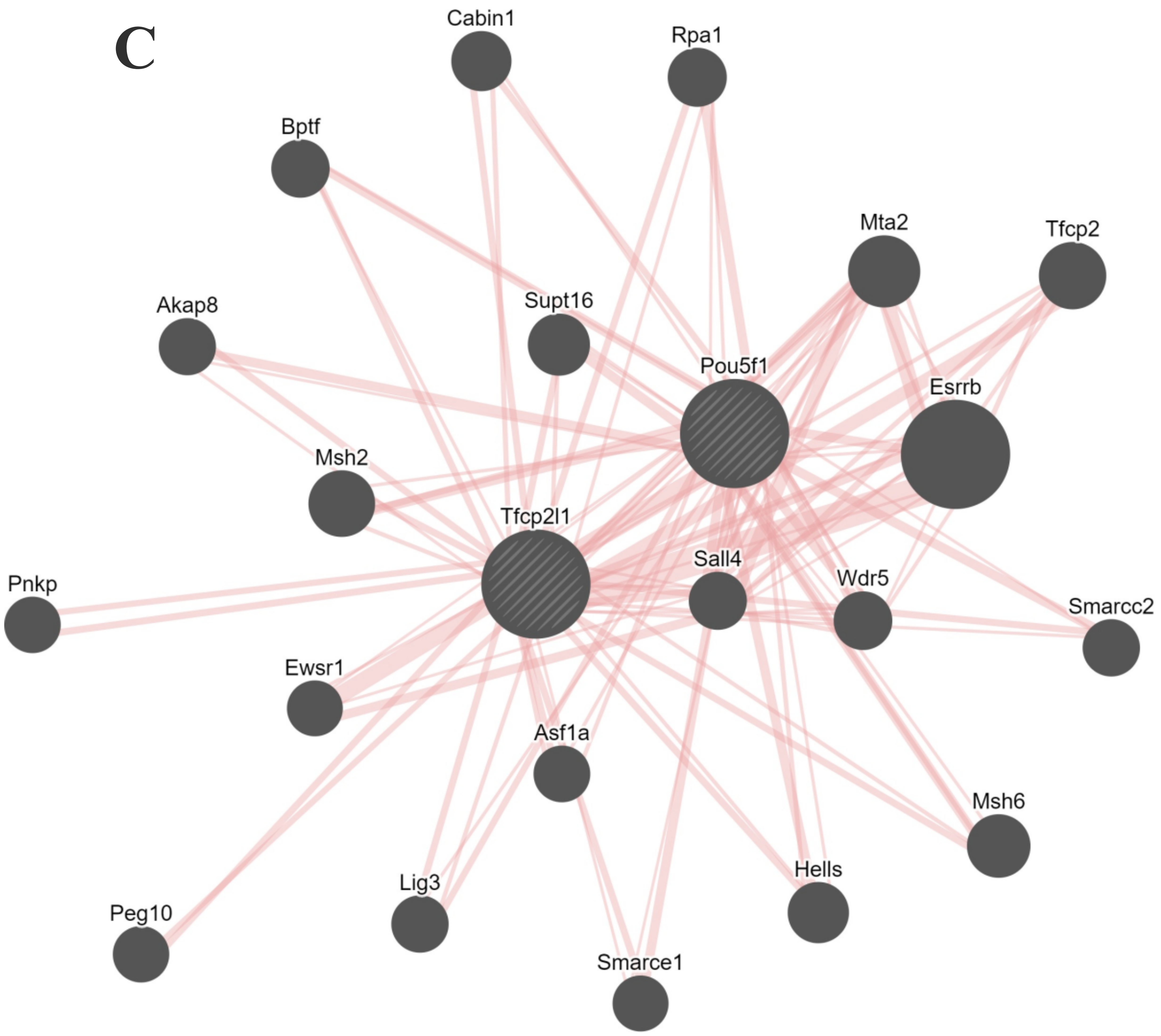

B

Sublist	Category	Term	RT	Genes	Count	%	P-Value	Benjamini
☐	INTACT	81879:transcription factor CP2-like 1(Tfcp2l1)	RT		18	7.3	8.8E-12	2.4E-9
☐	INTACT	217031:transcriptional adaptor 2A(Tada2a)	RT		8	3.2	4.3E-7	5.9E-5
☐	INTACT	14534:K(lysine) acetyltransferase 2A(Kat2a)	RT		8	3.2	1.6E-5	1.4E-3
☐	INTACT	18999:POU domain, class 5, transcription factor 1(Pou5f1)	RT		9	3.6	2.1E-3	1.3E-1
☐	INTACT	66233:DNA methyltransferase 1-associated protein 1(Dmap1)	RT		4	1.6	2.7E-3	1.4E-1
☐	INTACT	17187:Max protein(Max)	RT		4	1.6	3.6E-3	1.5E-1
☐	INTACT	107951:cyclin-dependent kinase 9 (CDC2-related kinase)(Cdk9)	RT		7	2.8	4.6E-3	1.6E-1
☐	INTACT	19045:protein phosphatase 1, catalytic subunit, alpha isoform(Ppp1ca)	RT		4	1.6	9.0E-3	2.7E-1
☐	INTACT	19821:ring finger protein 2(Rnf2)	RT		5	2.0	9.1E-3	2.4E-1

Figure S4 Interaction analysis of specific proteins. Related to Figure 5.
(A) Tgfbr1 physically interact with 8 genes according to the analysis with DAVID Bioinformatics Resources 6.8.
(B) The protein interactions with these genes were analyzed with DAVID Bioinformatics Resources 6.8.
(C) 113 relationships were observed among Tfcp2l1, Oct4 and 20 related genes according to the analysis with DAVID Bioinformatics Resources 6.8.
