# Supplementary material for: Transcriptional defects and reprogramming barriers in somatic cell nuclear reprogramming as revealed by single-embryo RNA sequencing
Source: BMC Genomics. 2018 Oct 10;19:734. doi: 10.1186/s12864-018-5091-1 (PMC6180508; doi:10.1186/s12864-018-5091-1)
Supplement: Supplementary file 1 — Gene expression in each sample. (PDF 220 kb) [file 12864_2018_5091_MOESM1_ESM.pdf]

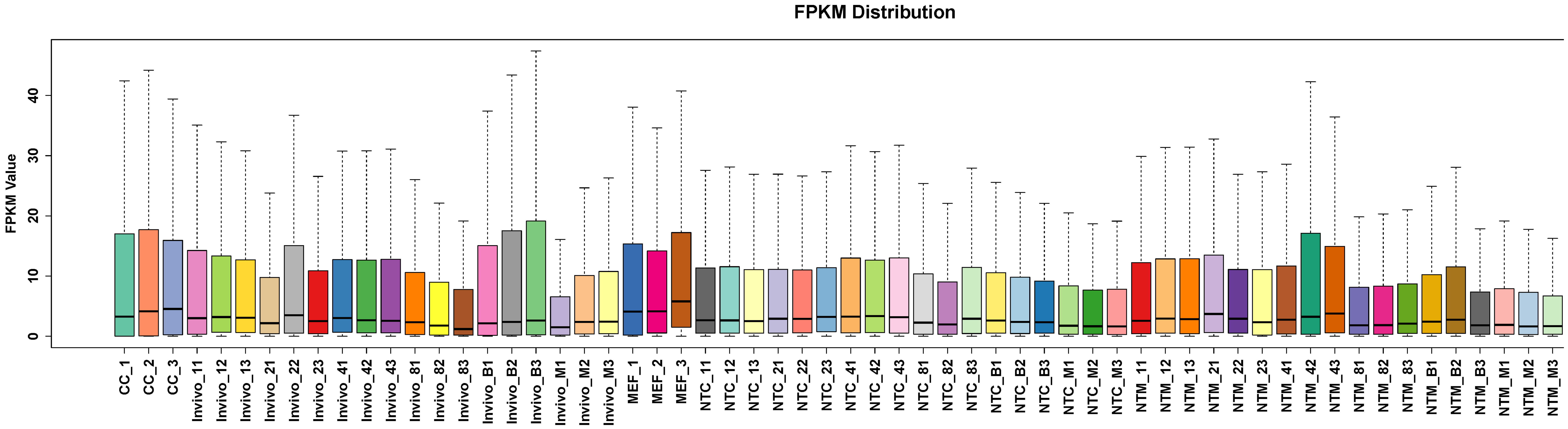

Figure S1 The gene expression of each sample. Related to Figure 1.
CC_1 means the first sample of CC group, CC_2 means the second sample of CC group. Invivo_11 means the first sample of Invivo_1 group, Invivo_12 means the second sample of Invivo_1 group. Invivo_21 means the first sample of Invivo_2 group, Invivo_22 means the second sample of Invivo_2 group. The rest can be done in the same manner.
