# Supplementary material for: Transcriptional defects and reprogramming barriers in somatic cell nuclear reprogramming as revealed by single-embryo RNA sequencing
Source: BMC Genomics. 2018 Oct 10;19:734. doi: 10.1186/s12864-018-5091-1 (PMC6180508; doi:10.1186/s12864-018-5091-1)
Supplement: Supplementary file 5 — Ensembl gene IDs of selected cluster genes. (PDF 1632 kb) [file 12864_2018_5091_MOESM5_ESM.pdf]

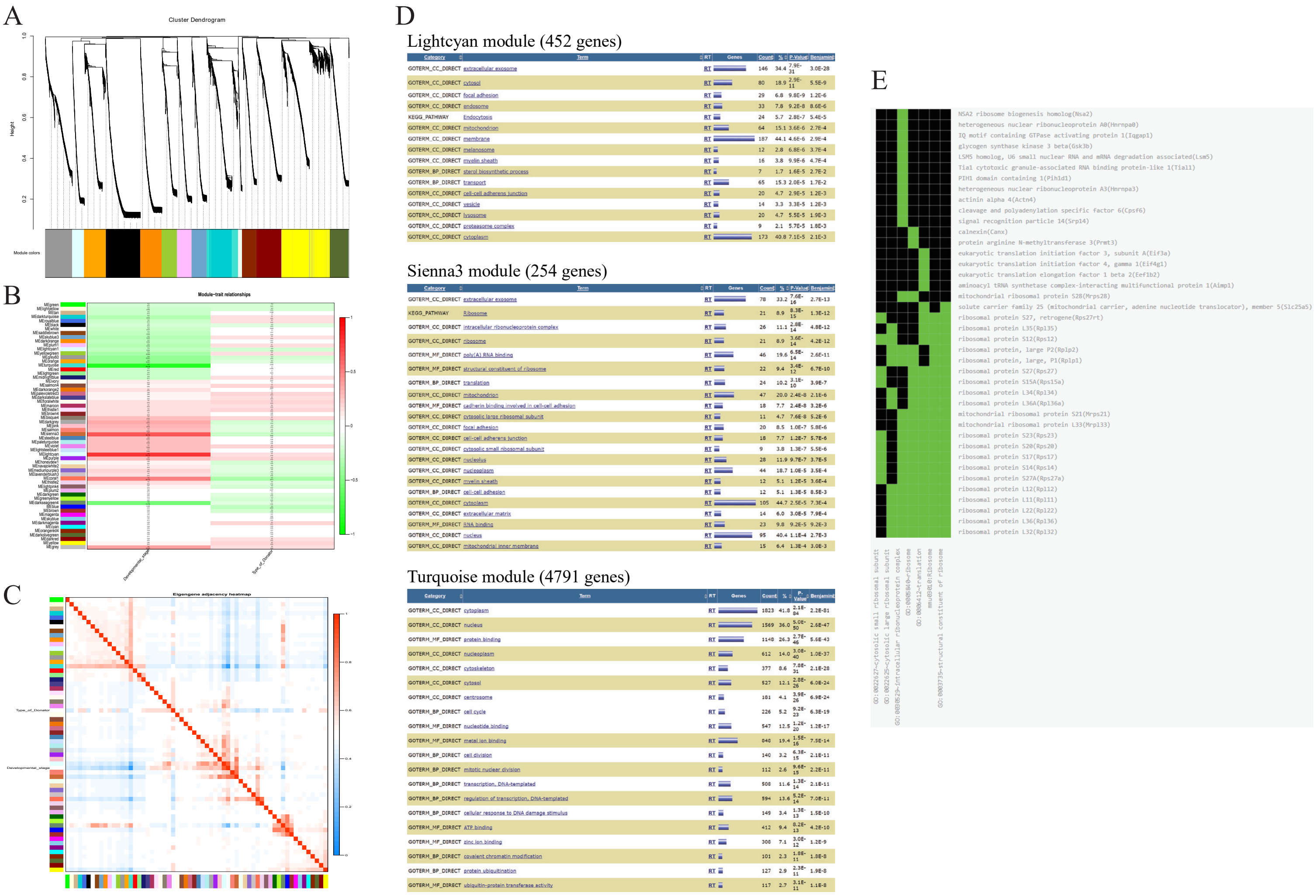

Figure S5 Weighted gene co-expression network analysis (WGCNA) of embryonic samples, Related to Figure 2.

(A) Cluster dendrogram. In this figure, genes are clustered according to their correlation, and different colors are used to mark the classes. Different colors in the figure represent different categories (modules).

(B) Correlation heat maps based on the correlation between modules and embryonic characteristics. The value represents correlation, and the p value in parentheses, the darker the color, the higher the correlation.

(C) Eigengene adjacency heatmap. The redder the color, the greater the correlation between the two modules.

(D) GO results of the genes in Lightcyan, Sienna3 and Turquoise modules.

(E) Enrichment analysis in Sienna3 module. The horizontal coordinate is the terms of GO or KEGG. The ordinate is the name of the enriched gene. Green squares represent the gene belong to the term, and the dark squares represent the gene not belong to the term.

The same color markers in A, B and C represent the same modules.
