# Supplementary material for: Transcriptional defects and reprogramming barriers in somatic cell nuclear reprogramming as revealed by single-embryo RNA sequencing
Source: BMC Genomics. 2018 Oct 10;19:734. doi: 10.1186/s12864-018-5091-1 (PMC6180508; doi:10.1186/s12864-018-5091-1)
Supplement: Supplementary file 8 — Spliceosome KEGG pathway in the in vivo, NTC and NTM groups. (PDF 231 kb) [file 12864_2018_5091_MOESM8_ESM.pdf]

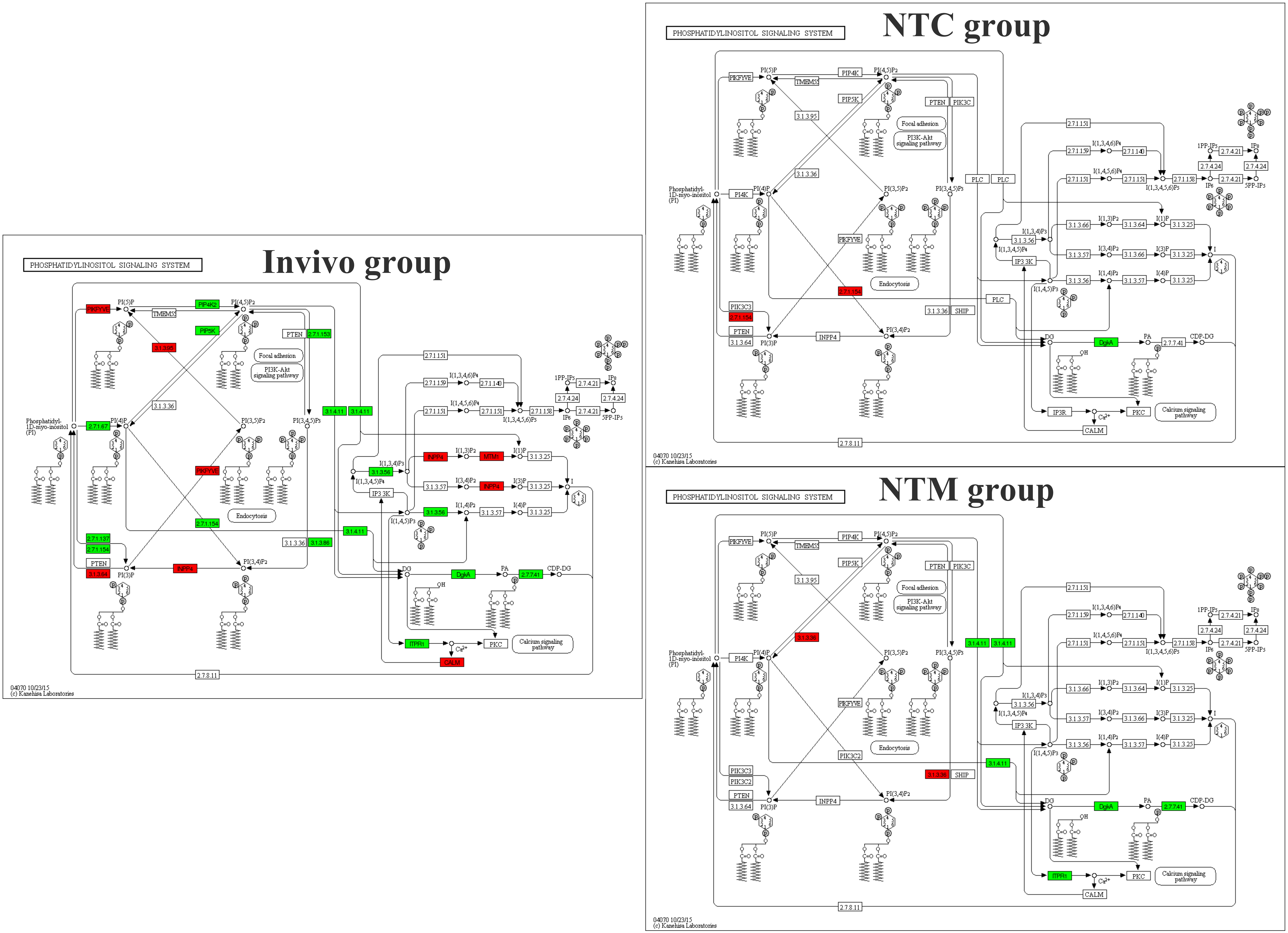

Figure S3 Spliceosome KEGG pathway of Invivo, NTC and NTM group. Related to Figure 4.
All five snRNP genes generate serious errors during transcription in the NT groups. Green means down-regulated when 2-cell embryos grow up to 4-cell embryos. Red means up-regulated.
